# Unexpected findings: loss of corneal endothelial cells in Uygur patients with exfoliation syndrome

**DOI:** 10.1007/s10792-024-02913-4

**Published:** 2024-02-13

**Authors:** Yinu Ma, Qin Li, Yue Dong, Xianglong Yi

**Affiliations:** Department of Ophthalmology, The First Affiliated Hospital of Xinjiang Medical University, Ürümqi, 830011 Xinjiang Uygur Autonomous Region China

**Keywords:** Anterior segment parameters, XFS, XFG

## Abstract

**Purpose:**

This study aimed to investigate anterior segment parameters in patients with exfoliation syndrome (XFS) and exfoliation glaucoma (XFG).

**Methods:**

The study adopted a retrospective case series design, involving a total of 56 patients (112 eyes) with unrelated XFS/XFG (XFS: 26 patients/60 eyes; XFG: 30 patients/44 eyes) and 100 age-related cataract cases as the control group (200 eyes). The participants were evaluated at the ophthalmology department of the First Affiliated Hospital of Xinjiang Medical University. Clinical data, including eye axial length, anterior chamber depth, white-to-white distance, central corneal thickness, and corneal endothelial cell density (ECD), were collected for statistical analysis.

**Results:**

ECD exhibited a significant difference between the XFS/XFG and age-related cataract groups (*P* < 0.001), while the remaining indexes did not show statistical differences (*P* > 0.05). Ocular parameters in patients with XFS and XFG were distinct from those in age-related cataract cases, with consistent results. Notably, there were no statistically significant differences between XFS and XFG patients.

**Conclusions:**

ECD is reduced in XFS/XFG patients compared with age-related cataract subjects. It is crucial to remain vigilant to enhance surgical safety in XFS/XFG patients and prevent complications proactively.

## Background

Exfoliation syndrome (XFS) is a condition wherein the extracellular matrix (ECM) undergoes changes, affecting individuals of various ages. It is marked by the continuous buildup of abnormal fibrils in intra and extraocular structures [1, 2]. This condition impacts 10–20% of the elderly population, leading to a higher incidence among the elderly and glaucoma patients [3]. In China, 5.1% of Kashi Uygur residents [4], and 2.2% and 9.5% of Kuche Uygur individuals aged 60 and 80 years or more, respectively, exhibit XFS [5].

XFS gives rise to eye complications, such as exfoliation glaucoma (XFG), and heightens the risk of unsuccessful intraocular surgery due to zonular weakness. XFS endotheliopathy is a gradually advancing condition affecting the corneal endothelial layer, resulting in early corneal endothelial cell decompensation and potentially leading to serious bullous keratopathy [6–8]. Numerous studies have examined the clinical data of XFS cases, investigating the characteristics of their anterior segment parameters. However, there is no consensus in the existing literature. To enhance the quality of available data and consider the prevalence of this condition, we conducted an assessment of anterior segment characteristics in Uyghur XFS/XFG cases.

## Methods

### Patients

The inclusion criteria for this study comprised a diagnosis of XFS, confirmed by the identification of exfoliation materials on the anterior lens capsule or pupil margin in one or both eyes following pupil dilation. A diagnosis of XFS was further substantiated by intraocular pressure (IOP) being less than 21 mmHg and the absence of glaucomatous optic neuropathy.

XFG was diagnosed based on the aforementioned exfoliation features, along with the following criteria: (1) IOP equal to or greater than 22 mmHg in one or both eyes; (2) diffused glaucomatous enlargement of the cup changes in the optic disc; (3) visual field loss attributed to glaucoma [9]. Individuals exhibiting causative factors for secondary glaucoma, such as uveitis, pigment dispersion syndrome, and iridocorneal endothelial syndrome, were excluded. Uygur residents aged 45 years and above were included in the study.

Control cases were enrolled based on the absence of exfoliation materials on the anterior lens capsule or pupil margin in both eyes after pupil dilation. Additional criteria included no diffused glaucomatous enlargement of the cup changes in the optic disc, normal IOP, vision consistent with cataract, no family history of glaucoma, and the absence of eye pathology, except for low refractive errors (It refers to the absence of axial refractive problems, but may cause low refractive error by cataracts). Participants were unrelated and underwent comprehensive ophthalmic examinations.

Exclusion criteria included: (1) a history of other eye diseases and previous ophthalmic surgeries; (2) refusal to continue cooperation by the selected subjects.

The present trial received approval from the Ethics Committee for Human Research of the First Affiliated Hospital of Xinjiang Medical University, China, in accordance with the Declaration of Helsinki. All participants provided signed informed consent.

Clinical data were collected from the hospital case system, encompassing eye axial length (AL), anterior chamber depth (ACD), white-to-white distance (W–W), central corneal thickness (CCT), and corneal endothelial cell density (ECD) for Uyghur patients with XFS/XFG and age-related cataract patients who visited the ophthalmology department of the First Affiliated Hospital of Xinjiang Medical University between May 2014 and November 2021.

Zeiss Optical Biometry IOLMaster 500, Germany: the subject sat without the need for surface anesthesia. The head was positioned closely in the headrest. The subject focused on the reticle. Measurements of AL of the eye, ACD, and W–W distance were conducted by the same experienced staff member. Five consecutive measurements were taken for each patient. The average value of these measurements was recorded to generate a comprehensive report.

Topcon SP-2000P Corneal Endothelial Cell Counter, Japan: the same operating technician examined both groups of patients. Each examination was repeated three times. The mean value of the three examinations was utilized for the analysis of changes in corneal ECD and CCT.

Data analysis was performed using SPSS 19.0 (SPSS, USA). Baseline data were compared using the χ^2^ test. Measures were presented as mean ± standard deviation and compared using the *T*-test, with *P* < 0.05 considered indicative of statistical significance.

## Results

A total of 56 Uygur patients with XFS/XFG (112 eyes) were included, comprising 44 XFG and 60 XFS eyes (data unavailable for 8 eyes). Additionally, 100 Uygur control patients with age-related cataracts (200 eyes) were enrolled. The study and control group had mean ages of 71.92 ± 5.81, 71.20 ± 4.76, and 71.01 ± 0.80 years, respectively (*P* > 0.05). The case group exhibited a predominance of male patients (*χ*^2^ = 17.45, *P* < 0.001), with 44 (78.57%) male and 12 (21.43%) female XFS/XFG patients. Meanwhile, the control group consisted of 44 (44%) male and 56 (56%) female participants (Table 1).

**Table 1:** Baseline patient features

	XFS	XFG	Control	*t*	*P*
	*n* = 26	*n* = 30	*n* = 100		
Age (years), mean ± SD	71.92 ± 5.81	71.20 ± 4.76	71.01 ± 0.80	1.53	0.129
				0.38	0.701
				0.51	0.612
Gender (M/F), *n* (%)	44 (78.57%)/12 (21.43%)		44(44%)/56(56%)	17.45	0.000

Baseline data were compared by the *χ*^2^ test. Measures were represented by mean ± standard deviation and compared by the *T*-test

M, male; F, female

We conducted a statistical analysis of eye parameters, including AL, ACD, CCT, and ECD, in both groups. A statistically significant difference was observed in ECD between the XFS/XFG and the control group, whereas the remaining indices did not exhibit significant differences. Further analysis of ocular parameters in XFS and XFG patients, distinct from those with age-related cataracts, revealed consistent findings. Notably, there were no significant differences between XFS and XFG patients (Table 2).

**Table 2:** Eye parameters in the XFS/XFG and the control group

Parameter	Case (*n*)	Value (*n*)	*t*	*P*
AL	XFS	22.91 ± 1.27 (51)	1.65	0.102^p1^
	XFG	23.35 ± 1.23 (39)	0.57	0.568^p2^
	XFS/XFG	23.15 ± 1.26 (97)	2.04	0.042^p3^
	Control	23.00 ± 0.92(195)	1.16	0.249^p4^
ACD	XFS	2.80 ± 0.50 (41)	0.00	1.000^p1^
	XFG	2.80 ± 0.61 (35)	0.38	0.703^p2^
	XFS/XFG	2.81 ± 0.55 (83)	0.34	0.732^p3^
	Control	2.83 ± 0.45(199)	0.32	0.751^p4^
W–W	XFS	11.45 ± 0.46 (41)	0.84	0.406^p1^
	XFG	11.54 ± 0.47 (34)	0.14	0.887^p2^
	XFS/XFG	11.50 ± 0.45 (82)	1.31	0.191^p3^
	Control	11.44 ± 0.40(199)	1.1	0.272^p4^
CCT	XFS	519.02 ± 52.90 (49)	0.01	0.990^p1^
	XFG	518.90 ± 33.52 (41)	1.02	0.307^p2^
	XFS/XFG	518.80 ± 43.72 (97)	1.05	0.295^p3^
	Control	526.44 ± 43.43(199)	1.42	0.157^p4^
ECD	XFS	2287.45 ± 559.80 (49)	0.11	0.914^p1^
	XFG	2275.93 ± 419.59 (40)	3.42	0.001^p2^
	XFS/XFG	2270.99 ± 494.24 (96)	3.75	< 0.001^p3^
	Control	2492.32 ± 312.59(196)	4.65	< 0.001^p4^

Measures were represented by mean ± standard deviation and compared by the *T*-test

XFS, exfoliation syndrome; XFG, exfoliation glaucoma; AL, axial length; ACD, anterior chamber depth; W–W, white-to-white distance; CCT, central corneal thickness; ECD, corneal endothelial cell density

^p1^XFS: XFG; ^p2^XFS: control; ^p3^XFG: control; ^p4^XFS/XFG: control

## Discussion

XFS exerts a comprehensive impact on the entire eye, with a notable emphasis on the anterior segment due to the accumulation of exfoliation material (XFM). Independently, it is linked with various disorders, including a small pupil, cataract, and zonular laxity. XFG, as the most common and severe associated disease, demonstrates heightened aggressiveness in comparison to primary open-angle glaucoma (POAG) [10–13]. These distinctive features render XFS patients particularly vulnerable to corneal endothelium-related complications during cataract phacoemulsification surgery. Our study represents the first exploration of the anterior segment parameters before operation in Xinjiang Uygur, China, for XFS/XFG patients. It reveals a statistically significant reduction in corneal ECD in Uyghur XFS/XFG patients compared to older cataract patients. This underscores the importance of assessing corneal status preoperatively and implementing intraoperative corneal endothelial protective measures.

Vaiciuliene et al. [14] conducted a review, noting that Tomaszewski et al. had previously used specular microscopy to assess ECD in individuals with 133 eyes with XFS syndrome (65 with glaucoma, 68 without glaucoma) and 84 eyes without XFS syndrome. In comparison to the control group, the XFS group exhibited a significantly lower ECD (2297 ± 359 cells/mm^2^ vs. 2503 ± 262 cells/mm^2^). This study also examined the ECD change in the XFG group, revealing that although there was no statistically significant difference between the XFS and XFG groups, the ECD in the XFG group (2241 ± 363 cells/mm^2^) was significantly lower than that in the control group [15]. Kristianslund et al. indicated that, before surgery, there was no significant difference in ECDs between the XFS and control groups [16]. Ucar et al. [17] similarly found no difference in ECD between XFS and cataracts before their investigation.

Various studies have assessed anterior ocular segment parameters in both XFS and XFG patients, yielding diverse findings. Kaygisiz et al. [18] compared these parameters among XFS, XFG, and normal subjects, finding no differences in anterior segment indexes between the XFS and XFG groups. However, in another report, corneal biomechanical indexes, including corneal hysteresis (CH), corneal resistance factor (CRF), and CCT, differed in XFS cases compared to healthy controls, with more pronounced changes in XFG cases [19]. In Turkey patients, those with XFG and XFS exhibited greater lens thickness, increased ACD, and reduced CCT compared to normal subjects [18]. Contrarily, our study found no significant difference in CCT between XFS/XFG and control patients, while a clear and significant difference was observed in corneal ECD, without distinction between the XFS and XFG groups. ECD, being a crucial indicator reflecting corneal condition, warrants special consideration in individuals with chronic eye disorders like recurrent uveitis, glaucoma, and PEX. It is also noteworthy in cases of IOL dislocation, especially prior to ophthalmological interventions [14]. We hypothesize that factors such as the accumulation of XFM may influence anterior chamber indexes during the progression from XFS to XFG, altering anterior chamber structures.

The outcomes of these studies have shown less consistency, and our endeavor is to contribute additional data to the clinical profile of individuals affected by XFS/XFG. Another study suggested elevated incidence rates for glaucoma (77.4%), cornea guttata (45.2%), and XFG (16.1%) in cases with a short AL [20]. However, our data showed no difference in AL between XFS/XFG and cataract patients. There were also no differences in ACD and W–W distance. This may be attributed to the limited sample size, necessitating a broader case pool, balanced gender representation, and preferably multicenter clinical studies for more robust and convincing data. This study underscores the importance of careful patient selection and adequate sample size, with a preference for using the normal population as a reference for comparison.

## Conclusions

In this study, corneal ECD was found to be reduced in patients with XFS and XFG when compared to age-related cataract subjects. Despite the lower ECD values observed, all participants maintained values greater than 2000/mm^2^. Advancements in equipment and surgical techniques have significantly reduced the risk of corneal decompensation following cataract surgery in XFS patients. However, it is crucial to remain vigilant in order to enhance surgical safety in individuals with XFS/XFG and proactively address potential complications before they arise.

## Data Availability

All data are available upon request from the corresponding author at yixianglong1010@163.com.
